# Awareness and Use of Folic Acid Among Pregnant Women in Western Ukraine: A Pilot Study

**DOI:** 10.3390/ijerph23030339

**Published:** 2026-03-08

**Authors:** Kateryna Hlushko, Oksana Boyarchuk

**Affiliations:** Department of Pediatrics, I. Horbachevsky Ternopil National Medical University, 46001 Ternopil, Ukraine; boyarchuk@tdmu.edu.ua

**Keywords:** folic acid, pregnant women, awareness, neural tube defects, prevention

## Abstract

**Highlights:**

**Public health relevance—How does this work relate to a public health issue?**

- This pilot study addresses an important public health concern in Ukraine: persistently high rates of neural tube defects (NTDs), which remain among the highest in Europe.
- It identifies a substantial gap between recommended and actual folic acid (FA) use, with low preconception supplementation (25.3%) compared to high post-conception initiation (80.0%), reflecting missed opportunities for effective NTD prevention.

**Public health significance—Why is this work of significance to public health?**

- Based on cross-sectional data, the study provides preliminary evidence on determinants of FA supplementation, demonstrating that preconception counseling, pregnancy planning, and prior supplementation behavior strongly influence preconception intake.
- The findings show that knowledge alone does not translate into preventive behavior, suggesting limitations in current NTD prevention strategies in Ukraine.

**Public health implications—What are the key implications or messages for practitioners, policy makers and/or researchers in public health?**

- Results support strengthening preconception counseling and promoting folic acid supplementation among women of reproductive age.
- Implementation of national food fortification policies with folic acid and conduction of larger, population-based studies to develop evidence-based prevention strategies in Ukraine are recommended.

**Abstract:**

Objective: Neural tube defects remain a significant global health concern. This pilot study assessed folic acid (FA) knowledge and supplementation practices among pregnant women in Ukraine. Methods: A cross-sectional survey was conducted among 95 pregnant women who completed a 22-item self-administered questionnaire assessing FA awareness and socio-demographic characteristics. Results: Although 76.8% of participants reported planned pregnancies, only 25.3% used FA before conception, while 80.0% initiated supplementation after pregnancy recognition (*p* < 0.05). In bivariate logistic regression analysis, preconception counseling (OR = 7.7, 95% CI: 2.37–24.85), planned pregnancy (OR = 9.7, 95% CI: 1.22–76.25), previous FA supplementation (OR = 3.11, 95% CI: 1.20–8.33), and increasing maternal age (OR = 1.09 per year, 95% CI: 1.00–1.19) were significantly associated with preconception FA use. Sociodemographic factors were not significant predictors. For FA use during pregnancy, only previous supplementation remained significant (OR = 4.10, 95% CI: 1.10–14.29). Awareness of recommended FA use before (35.8%) and during pregnancy (48.4%) and knowledge of neural tube defect prevention (20.0%) were low and not associated with supplementation behavior. Conclusions: A substantial gap exists between recommended and actual FA use, particularly before conception. Strengthening preconception counseling may improve timely supplementation.

## 1. Introduction

Neural tube defects (NTDs) continue to be a significant and prevalent medical issue in many countries. They are congenital defects that occur within the first 28 days of fetal development, often before a woman is even aware of her pregnancy. This anomaly is the result of an incomplete closure of the neural tube in the embryo, resulting in varying consequences depending on the location and severity of the defect [1]. NTDs are primarily sporadic and have a multifactorial etiology, including both environmental factors and genetic or chromosomal abnormalities (which account for less than 10% of all cases). The main environmental factor associated with NTDs is folic acid (FA) deficiency in pregnant women [2,3]. Other potential causes include maternal obesity, diabetes, fever during pregnancy, pesticide exposure, smoking, a previous pregnancy affected by NTDs, and intake of antiepileptic medications (valproic acid increases the chances of NTD development by about tenfold) [2,3].

Cranial and open spinal defects are potentially the most difficult and can result in the death of a child (such as anencephaly and encephalocele), or lead to neurological impairment, skeletal deformities, and neurogenic bladder and/or bowel disorders, which are just some of the main health concerns for these patients. Despite improvements in surgical treatment of patients with spina bifida, they often experience disabilities and require ongoing multidisciplinary care [1,4]. This not only places a significant financial burden on the medical system and families, but also leads to social and psychological challenges for both the children and their families, which all are particularly crucial for Ukrainian spina bifida patients due to the onset of war [5,6].

The prevalence of NTDs varies globally, with an average prevalence of about 20 cases per 10,000 births, and depends on factors such as climate, nutritional habits, nationality, consanguinity rate, and mandatory folic acid food fortification [1,3,7,8,9]. For example, a review of studies on folate status in women of reproductive age showed that the prevalence of folate deficiency, which is associated with megaloblastic anemia, was higher than 20% in countries with lower income economies, compared to less than 5% in countries with higher income. In addition, the prevalence of folate insufficiency, which increases the risk of NTD-affected pregnancies, was higher than 40% in most countries [9]. Findings from a study conducted among Ukrainian children revealed that 32.9% of them were deficient in folate, while children’s consumption of food rich in FA was more than two times lower than the recommended amount for their age [10]. Strong evidence supports the effectiveness of periconceptional folic acid supplementation in preventing NTDs. An umbrella review of systematic reviews and meta-analyses including 296,816 participants reported that prenatal FA or multivitamin supplementation was associated with a 57% reduction in NTD risk globally (pooled effect = 0.43, 95% CI: 0.29–0.58). The protective effect was more pronounced for FA supplementation alone, which was associated with a 77% reduction in NTD risk, compared with a 37% reduction observed for multivitamin use [11]. The implementation of mandatory folic acid fortification has resulted in a significant decrease in the prevalence of NTDs in countries like Canada, where the rate decreased from 1.58 per 1000 births before fortification to 0.86 per 1000 births after fortification, and in the USA, where it reduced from 0.76 per to 0.56 per 1000 births. However, in Europe, where folic acid fortification is not mandatory, there has been no significant change [7]. In Ukraine, fortification of wheat flour and other food products with folic acid is not mandatory and is currently performed mainly for export purposes.

According to “OMNI-Net Ukraine” data, the international non-profit, non-governmental charitable fund, the prevalence of NTDs in Ukraine was 2.04 per 1000, the highest reported in Continental Europe [2,12]. Additionally, researchers observed regional differences in NTD prevalence between areas affected by the Chernobyl nuclear disaster (1986) and those that were not. Specifically, the prevalence of NTDs in radioactively contaminated regions was 2.3 per 1000 births, compared with 1.75 per 1000 in non-affected areas [2,13,14]. Furthermore, the EUROCAT report showed that the prevalence of NTDs in Ukraine was the second highest in Europe (1.80 per 1000), after Bulgaria (2.07), and was followed by Germany (1.63) and the UK (1.2–1.41) [7]. In comparison, Portugal (0.43) and Italy (0.56) had the lowest rates [7].

Recent studies also suggests that maternal micronutrient supplementation during pregnancy may also influence broader child health outcomes beyond neural tube defect prevention. A large population-based cohort study from the Netherlands including 3937 children demonstrated that maternal vitamin supplementation, including folate and multivitamins, was associated with improved neurodevelopmental outcomes and fewer symptoms of attention-deficit hyperactivity disorder and autism spectrum disorder in offspring, partly mediated by differences in brain morphology [14]. Adequate maternal folate status plays a critical role not only in fetal development but also in overall pregnancy outcomes. Recent evidence from China shows that higher maternal plasma and red blood cell folate concentrations are associated with overall favorable pregnancy outcomes, whereas metabolic markers of vitamin B deficiency, such as elevated homocysteine and methylmalonic acid, are associated with increased risk of adverse pregnancy outcomes [15].

Hence, providing adequate maternal FA nutritional status is one of the most effective ways to prevent NTD development as well as other adverse pregnancy outcome. To achieve this, major international health organizations recommend routine periconceptional FA supplementation for all women of reproductive age. According to the World Health Organization (WHO), women should consume 400 μg (0.4 mg) of folic acid daily to reduce the risk of NTDs [16]. Additionally, the WHO provides biomarker-based guidance, recommending a red blood cell folate concentration of at least 906 nmol/L at the population level to achieve optimal NTD prevention [16]. Achieving adequate folate status prior to conception is particularly important, as many pregnancies are unplanned and dietary intake alone may be insufficient to meet physiological requirements [16]. Similarly, the United States Centers for Disease Control and Prevention recommends that all women capable of becoming pregnant consume 400 μg of folic acid daily, beginning at least one month before conception and continuing during early pregnancy [17]. For women at increased risk of NTDs, including those with a previous affected pregnancy, higher doses of 4 mg daily are recommended under medical supervision [17]. European guidelines are consistent with these recommendations. For example, the United Kingdom National Institute for Health and Care Excellence advises women to take 400 μg of folic acid daily from before conception until the 12th week of pregnancy, with a higher dose of 5 mg daily recommended for women at increased risk [18]. In Ukraine, similar periconceptional recommendations for folic acid supplementation are applied.

In addition to taking supplements, eating foods that are rich in folate can also help maintain adequate folate levels. Some natural sources of folate include dark green leafy vegetables, legumes, citrus fruits, asparagus, broccoli, nuts, and beef liver [1,10,16,17,18].

Despite existing international recommendations, many women do not take folic acid before getting pregnant. Limited awareness, lack of preconception counseling, absence of mandatory food fortification programs in some countries, can contribute to insufficient folic acid intake among women of reproductive age.

This pilot study aimed to assess folic acid supplementation practices among pregnant women, including use before and during pregnancy, factors influencing supplementation behavior, and awareness of its role in fetal development.

## 2. Materials and Methods

### 2.1. Study Design and Setting

This pilot cross-sectional study was conducted between June and August 2023 among pregnant women attending a local maternity outpatient clinic in Ternopil, western Ukraine. Ternopil is a regional administrative center with an estimated population of approximately 217,987 residents (2025). The clinic provides routine antenatal care services to women from the city and surrounding areas. The study was designed to generate preliminary data on folic acid knowledge and supplementation practices to evaluate the need and rationale for a larger, population-based investigation in Ukraine.

### 2.2. Participants

Eligible participants were pregnant women at any stage of pregnancy (first, second, or third trimester) who attended the clinic during the study period and agreed to participate. Women who were unable to complete the self-administered questionnaire independently were excluded.

A total of 95 pregnant women aged 18–41 years participated in the study. The mean age was 28.6 ± 5.5 years. All participants provided informed consent prior to enrollment.

### 2.3. Data Collection Tool

Data were collected using a structured, self-administered 22-item questionnaire developed by the authors. The questionnaire included the following domains:

− Sociodemographic characteristics, including age, place of residence (urban/rural), and educational level;
− Anthropometric information, including self-reported pre-pregnancy body weight and height;
− Reproductive history, including gravidity, pregnancy planning status, number of previous pregnancies, presence of children, and family history of birth defects;
− Folic acid supplementation practices, including use before conception, timing of initiation during pregnancy, duration of intake, and previous use in earlier pregnancies;
− Knowledge and awareness of folic acid, including awareness of the necessity of FA before and during pregnancy, knowledge of recommended duration of FA supplementation, knowledge of its role in neural tube defect prevention;
− Awareness of dietary sources of folate was assessed using a two-step approach. Participants were first asked whether they were aware of food sources of folate (yes/no). Subsequently, all participants were provided with a predefined list of food items and asked to identify those they considered to be sources of folate. Multiple selections were allowed.

Questions were primarily multiple-choice or categorical (yes/no/I do not know). The questionnaire was not externally validated and was designed for exploratory assessment in this population.

### 2.4. Variables and Grouping

For comparative analyses, participants were categorized according to place of residence and educational level. Place of residence was classified as urban (*n* = 79; 83.2%) or rural (*n* = 16; 16.8%). Educational level was dichotomized into higher education (master’s degree; *n* = 66; 69.5%) and bachelor’s/secondary education (*n* = 29; 30.5%). Those who completed bachelor’s degree and school graduates were combined in together due to small sample of school graduates (*n* = 4).

Pre-pregnancy body mass index (BMI) was calculated using the standard metric formula, BMI = weight (kg)/height (m^2^), where weight was expressed in kilograms and height in meters squared. Pre-pregnancy BMI categories were classified according to World Health Organization criteria as follows: underweight (<18.5 kg/m^2^), normal weight (18.5–24.9 kg/m^2^), overweight (25.0–29.9 kg/m^2^), and obesity (≥30.0 kg/m^2^). BMI was analyzed both as a continuous variable and according to standard categories.

### 2.5. Statistical Analyses

Descriptive statistics were used to summarize participants’ socio-demographic and obstetric characteristics. Categorical variables are presented as frequencies and percentages, while continuous variables are expressed as means with standard deviations. Group comparisons were performed using the chi-square test for categorical variables and appropriate parametric or non-parametric tests for continuous variables, as applicable.

Given the limited number of outcome events, particularly for preconception folic acid intake, only bivariate logistic regression analyses were performed to examine the associations between FA supplementation (before and during pregnancy) and selected socio-demographic and obstetric characteristics. Multivariable logistic regression was not conducted to avoid model overfitting and unstable estimates.

Odds ratios (ORs) with corresponding 95% confidence intervals (CIs) were calculated to assess the strength and direction of associations. A *p*-value < 0.05 was considered statistically significant.

All statistical analyses were performed using the Statistical Package for Social Sciences (SPSS), version 23 (IBM Corp., Armonk, NY, USA).

## 3. Results

### 3.1. Sociodemographic Characteristics and Reproductive History

The majority of the surveyed women were in the middle of their reproductive years (57.9%), lived in urban areas (83.2%), held master’s degrees (69.5%) and stated middle family income (66.3%). Table 1 present general characteristic of recruited women including sociodemographic characteristics, pregnancy and reproductive history.

No significant differences were observed in sociodemographic or pregnancy-related characteristics according to place of residence. The only statistically significant association identified was between educational level and preconception body mass index category (χ^2^ = 6.73, *p* = 0.035). Women with a bachelor’s degree/secondary education were more likely to be overweight or obese compared with those holding a master’s degree. Specifically, overweight prevalence was 37.9% versus 24.2%, and obesity prevalence was 20.7% versus 7.6%, respectively. Notably, the proportion of obesity was nearly three times higher among women with bachelor’s/secondary education compared to those with higher university education.

### 3.2. Folic Acid Supplementation Practices

**Figure 1:**
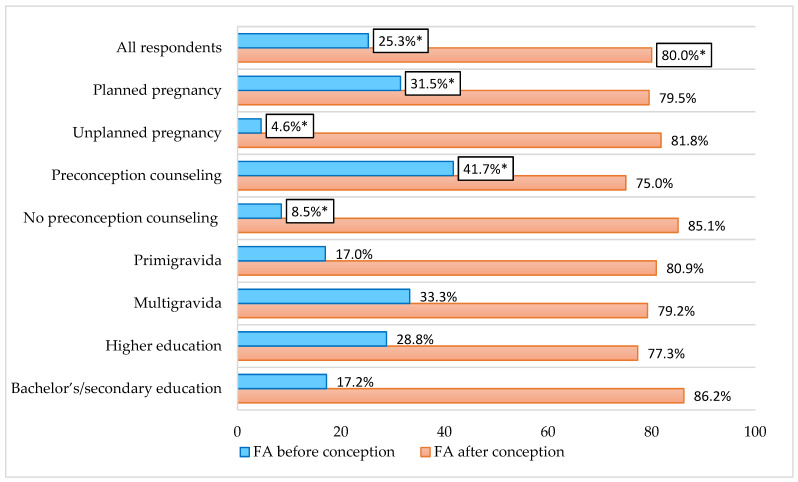
Preconception and post-conception folic acid supplementation among pregnant women (*n* = 95), stratified by selected demographic and obstetric characteristics. Values represent percentages. * *p* < 0.05 between subcategories.

Overall, 80.0% (*n* = 76) of respondents reported initiating FA supplementation after conception, whereas only 25.3% (*n* = 19) reported use prior to conception (*p* < 0.05). Preconception FA supplementation was significantly associated with pregnancy planning and receipt of preconception counseling. In contrast, no significant associations were observed with educational level or parity. Likewise, post-conception FA use did not differ significantly across sociodemographic or obstetric characteristics. Figure 1 illustrates the distribution of periconceptional folic acid intake among all participants, stratified by selected demographic and reproductive factors.

Additionally, women who reported preconception FA use were significantly older than those who did not (30.6 ± 4.5 years vs. 28.0 ± 5.6 years, Z = −2.3, *p* = 0.02). In contrast, no significant age differences were observed with respect to FA intake during pregnancy (28.7 ± 5.4 years among users vs. 28.4 ± 5.9 years among non-users, *p* > 0.05). Finally, preconception FA supplementation did not differ significantly according to place of residence.

Overall, 37.9% (*n* = 36) of participants reported having taken FA during a previous pregnancy, while 12.6% (*n* = 12) reported no prior FA use. The remaining 50.5% (*n* = 49) either had not been pregnant previously or did not recall prior FA intake.

The results of the bivariate logistic regression analysis for predictors of preconception FA supplementation are presented in Table 2.

These findings indicate that preconception supplementation was primarily influenced by counseling, pregnancy planning, prior supplementation behavior, and maternal age, rather than sociodemographic characteristics. Additionally, it was found that each additional pregnancy was associated with a 67% increase in the odds of preconception folic acid use (OR = 1.67, 95% CI: 1.01–2.77, *p* = 0.044), although the lower confidence limit suggests cautious interpretation.

The bivariate logistic regression analysis of predictors of folic acid use after conception is presented in Table 3. In contrast to preconception supplementation, few variables were significantly associated with FA intake after pregnancy recognition.

Additionally, it was found that pregnancy order was not significantly associated with folic acid use during pregnancy (OR = 1.24 per pregnancy, 95% CI: 0.68–2.26, *p* = 0.491).

These results suggest that, once pregnancy was recognized, folic acid use became widespread and largely independent of demographic and reproductive characteristics. In contrast, preconception supplementation was strongly influenced by planning, counseling, and previous behavior.

### 3.3. Knowledge and Awareness of Folic Acid

Most of the respondents, 84.2% (*n* = 80), stated that they had heard about FA, while significantly fewer, 61.1% (*n* = 58), knew that it was a vitamin (*p* < 0.05). Another 25.3% (*n* = 24) admitted that they did not know what it was, and 13.6% (*n* = 13) provided an incorrect definition (fatty acid, hormone, etc.).

**Figure 2:**
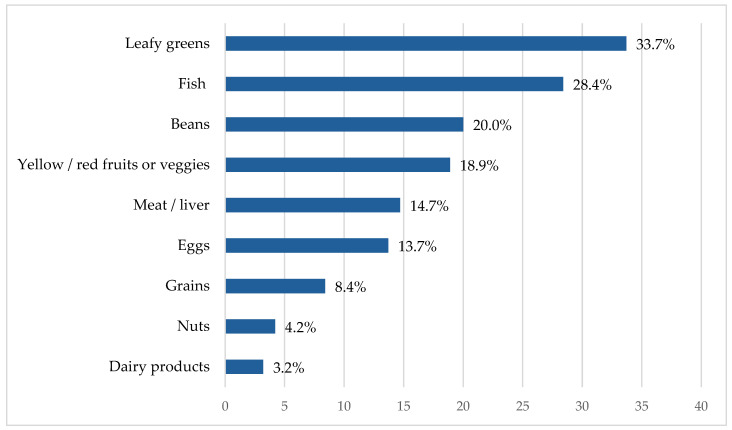
Respondents’ self-assessment regarding what is the main food sources of folates. Values represent percentages and represent the proportion of the total sample (*n* = 95).

The findings indicate limited knowledge among respondents regarding dietary sources of folic acid. Overall, 41.1% (*n* = 39) of participants reported that they knew dietary sources of folate, whereas 58.9% (*n* = 56) indicated that they did not. When asked to identify specific sources (multiple responses allowed), 33.7% identified leafy green vegetables (the most well-established natural source), 28.4% fish, 20.0% beans, and smaller proportions identified other food items. Figure 2 illustrates respondents’ self-assessment regarding what food is rich with folates.

There were no difference in respondents’ knowledge regarding food sources of FA depending on educational level or place of residence. In addition, 68.4% (*n* = 65) believed that they follow balanced nutrition, but this statement demands further assessment as women may have different ideas about proper nutrition.

Findings regarding women`s knowledge about FA’s impact on fetus health showed that majority of surveyed, specifically 80.0% (*n* = 76), were not aware about possibility of NTD development. At the same time, the majority of respondents (73.7%) acknowledged that FA deficiency could affect fetal health, while 26.3% reported that they “Did not know”; none of the participants responded “No.” When asked whether FA deficiency could cause disease in the fetus, 48.4% answered “Yes,” 3.2% answered “No,” and an equal proportion (48.4%) indicated that they “Did not know”. Awareness was lowest regarding the association between FA deficiency and birth defects: only 20.2% of respondents answered “Yes,” whereas 12.6% answered “No,” and 67.4% reported that they “Did not know”. These data did not differ based on participants’ education or residency.

Table 4 presents women’s awareness regarding necessity and duration of periconceptional FA supplementation overall in the total population and based on educational level.

Our findings indicated that only 35.8% (*n* = 34) of participants were aware of the need for FA supplementation before pregnancy, whereas 48.4% (*n* = 46) recognized its importance after conception; however, this difference was not statistically significant (*p* > 0.05).

Despite higher levels of awareness among women with higher education, knowledge regarding the necessity and timing of folic acid supplementation—including awareness of the adverse fetal effects associated with folic acid deficiency—did not translate into consistent or adequate folic acid intake in practice.

Finally, only one-third of those surveyed, or 35.8% (*n* = 34), admitted that they would consider taking FA for prevention. Another 20.0% (*n* = 19) denied this possibility, while approximately 44.2% (*n* = 42) were unsure about whether or not they would take it. These data did not differ based on women`s education or residency.

## 4. Discussion

### 4.1. Interpretation of Folic Acid Supplementation Practices

The results of this pilot study demonstrate a marked difference between preconception FA supplementation (25.3%) and its initiation after pregnancy recognition (80.0%). A previous large-scale study conducted in Ukraine between 2010 and 2014 among 4798 pregnant women reported substantially lower rates of both preconception (6.8%) and post-conception (47.4%) FA use [2]. Although overall supplementation rates appear to have improved over time, the pattern remains consistent: preconception use is markedly lower than post-conception initiation. In both studies, most women began FA supplementation following their first antenatal visit, typically occurring at 4–6 weeks of gestation or even later. This timing is suboptimal, as neural tube closure occurs during the early embryonic period (first 21–28 days), often before pregnancy is clinically recognized. Our results should be interpreted cautiously given the limited and predominantly urban sample size as well as single-center design; however, they suggest that delayed initiation of FA supplementation may remain an ongoing public health concern in Ukraine.

Our findings are also consistent with reports from other countries demonstrating low rates of preconception folic acid use despite high post-conception supplementation. Similar patterns have been observed in Ireland (30.2% preconception vs. 96.5% post-conception use) [19], Lebanon (33.6% vs. 93.9%) [20], Turkey (14.2% vs. 48.6%) [21], and Italy (23.5% vs. 54.9%) [22]. Studies from Japan (20.5% preconception use) [23] and China (25.8% correct preconception use) [24] likewise report suboptimal adherence to recommended periconceptional supplementation.

Evidence from low-income settings highlights the role of structural and sociodemographic factors. For example, a study conducted in Southwest Ethiopia reported that only 4.2% of pregnant women were aware of the need for folic acid intake during the preconception period [25]. In that study, husband’s formal education (AOR = 2.84), proximity to health facilities (AOR = 2.83), and attending four or more antenatal visits (AOR = 2.31) were independently associated with higher awareness. Although these determinants were not specifically evaluated in our study, such findings may indicate that access to healthcare services and household educational context may substantially influence preconception awareness and supplementation behavior. Overall, the evidence suggests that delayed initiation of folic acid supplementation may remain a global public health challenge, regardless of geographic or socioeconomic context.

### 4.2. Determinants of Pre- and Post-Conception Folic Acid Supplementation

In this exploratory pilot study, preconception FA use appeared to be more frequently observed among women reporting behavioral and reproductive factors rather than sociodemographic characteristics. Receiving preconception counseling (OR = 7.7), planned pregnancy (OR = 9.7), and previous FA supplementation (OR = 3.11) were significantly associated with preconception use in bivariate analysis. Increasing maternal age showed a modest association, while multigravidity demonstrated borderline statistical significance. In contrast, educational level, place of residence, and BMI were not significantly associated with preconception FA use. Given the limited sample size, these associations should be interpreted with caution; however, they may suggest that intentional reproductive planning and prior engagement with healthcare services could influence supplementation behavior in our cohort of Ukrainian women.

Similar results have been reported in other international studies. For example, Italian data demonstrated that women who planned their pregnancy and attended a preconception health visit were substantially more likely to initiate FA supplementation before pregnancy (48.6% vs. 4.8%) [22]. A Polish study likewise identified several factors associated with higher likelihood of preconception supplementation, including older maternal age, higher education, marriage or cohabitation, lower parity, infertility treatment, and the presence of chronic disease [26]. In contrast to these findings, in our exploratory cohort multigravidity showed a possible association with preconception FA use, whereas other sociodemographic factors were not statistically significant. However, marital status, infertility treatment, and chronic disease were not assessed in our study.

Unlike our results, previous international studies have identified a broader range of determinants influencing FA intake, including educational level, place of residence, income, ethnicity, and BMI. For example, Linnell et al. (2022) reported that women in Ireland with obesity were less likely to meet the recommended higher-dose FA supplementation requirements [19]. In our study, however, no association was found between overweight/obesity and periconceptional FA intake. Although women with overweight or obesity in our cohort tended to have lower educational levels, education itself was not significantly associated with preconception or post-conception FA use. This discrepancy may reflect the relatively homogeneous and predominantly urban composition of our sample, as well as the limited statistical power inherent in a pilot study. Furthermore, BMI values were calculated from self-reported pre-pregnancy weight and height, which may be subject to recall bias and potential misclassification.

Importantly, prior supplementation behavior was significantly associated with both preconception and post-conception FA use. Women who had previously taken FA were approximately three times more likely to use it before the current pregnancy and four times more likely to use it after conception. This behavioral consistency suggests the importance of family planning, early counseling and preconception education.

In contrast to preconception supplementation, relatively few factors were associated with FA use after pregnancy recognition. Aside from previous FA supplementation, no sociodemographic or obstetric variables—including age, education, residence, pregnancy planning, gravidity, or BMI—were significantly associated with post-conception supplementation in bivariate analysis. This may indicate that once pregnancy is recognized, FA use becomes more common and less dependent on background characteristics, possibly reflecting routine antenatal counseling practices.

### 4.3. Knowledge and Awareness of Folic Acid Use, Role and Supplementation

Despite relatively low levels of awareness regarding recommended FA supplementation before (35.8%) and during pregnancy (48.4%), knowledge was not significantly associated with actual supplementation behavior in our study. Although women with higher education demonstrated somewhat better awareness (40.9% vs. 24.1% before conception and 51.5% vs. 41.4% after conception), this did not correspond to higher supplementation rates. Similar discrepancies between knowledge and behavior have been observed among Ukrainian medical students, where high awareness (86.8%) did not result in consistent supplementation, which was reported by only 10% of respondents [27]. In contrast, a study among Polish women of childbearing age found that awareness of the protective effect of FA against NTDs significantly increased the likelihood of preconception supplementation (OR = 4.58) [26], suggesting that the relationship between knowledge and behavior may vary across settings.

Further evidence from Panasiuk et al. (2023) showed that only 12% of surveyed female students reported preventive FA intake, despite moderate awareness regarding NTD formation (52%), recommended timing (42%), dosage (34%), and dietary sources such as green leafy vegetables (55%) [28]. In comparison, awareness in our cohort was lower: only 20.0% recognized the role of FA deficiency in NTD development, and 33.7% identified leafy greens as a dietary source of folate. Although 35.8% of participants indicated that they would consider taking FA for prevention, intention does not necessarily translate into actual supplementation.

Overall, our findings may suggest that knowledge alone is not sufficient to ensure appropriate supplementation behavior. Behavioral and structural factors—such as preconception counseling, pregnancy planning, and prior supplementation experience—appeared more frequently associated with FA use than awareness measures in this exploratory analysis. Notably, the low level of awareness regarding FA’s role in NTD prevention (20.0%) observed in our study is comparable to data from a European survey involving 18 countries, which reported a 17% awareness rate [29]. Although these findings should be interpreted cautiously given the limited sample size, they may indicate a knowledge gap that warrants attention through systematic public health strategies rather than reliance on individual awareness.

## 5. Limitations

This study has several limitations that should be considered when interpreting the findings. First, the relatively small sample size (*n* = 95) limits statistical power and may reduce the precision of the estimated associations. Accordingly, this investigation should be considered a pilot study, designed to provide preliminary data into folic acid knowledge and supplementation practices among pregnant women in Ukraine and assess need for improved design of future, larger-scale studies. Second, the study was conducted in an urban outpatient clinic, which may have introduced selection bias and limited the representativeness of the sample. Women from rural areas, those with lower socioeconomic status, and individuals from socially vulnerable groups may have been underrepresented, resulting in a relatively homogeneous study population. Third, data were collected using a self-administered questionnaire without direct researcher assistance, which may have contributed to recall bias, particularly regarding prior pregnancies, previous folic acid use and pre-pregnancy anthropometric data. Additionally, time constraints inherent to the outpatient setting may affect data completeness and accuracy. Despite these limitations, the study provides important preliminary evidence regarding current periconceptional folic acid use in Ukraine, as the most recent national assessment of supplementation was conducted between 2010 and 2014.

## 6. Conclusions

This pilot study demonstrated that folic acid supplementation before pregnancy remains substantially lower than supplementation initiated after pregnancy recognition among Ukrainian women. Preconception counseling, pregnancy planning, prior supplementation behavior, and increasing maternal age were associated with higher odds of preconception FA use, whereas sociodemographic characteristics such as educational level, place of residence, and BMI were not significant predictors. Notably, awareness of FA recommendations and knowledge of its role in preventing neural tube defects did not significantly influence actual supplementation behavior.

Strengthening preconception-counseling services and integrating systematic FA education into routine reproductive healthcare may improve early supplementation practices. Healthcare providers should emphasize the importance of daily FA intake prior to conception, alongside guidance on dietary folate sources. At the population level, consideration of mandatory folic acid food fortification may represent an effective strategy for reducing neural tube defects in Ukraine.

Given the exploratory nature of this pilot study and the limited sample size, larger population-based investigations including diverse socio-demographic groups are required to confirm these findings and further examine determinants of FA supplementation among Ukrainian women.

## Figures and Tables

**Table 1:** General characteristic of the respondents (*n* = 95).

Variable	Category	*n* (%)
Sociodemographic Characteristics		
Age (years)	18–24	23 (24.2)
	25–34	55 (57.9)
	≥35	17 (17.9)
Educational Level	Master’s degree	66 (69.5)
	Bachelor’s degree/school	29 (30.5)
Occupation	Student	7 (7.4)
	Housewife	26 (27.4)
	On maternity leave	35 (36.8)
	Employed	27 (28.4)
Residence	Urban	79 (83.2)
	Rural	16 (16.8)
Family Income Level (self-assessment)	Low	10 (10.5)
	Lower-middle	14 (14.7)
	Middle	63 (66.3)
	Upper-middle	8 (8.4)
Pregnancy and Reproductive History		
Pregnancy Planning	Planned	73 (76.8)
	Unplanned	22 (23.2)
Gravidity	Primigravida	47 (49.5)
	Multigravida	48 (50.5)
Trimester of Pregnancy	First trimester	6 (6.3)
	Second trimester	45 (47.4)
	Third trimester	44 (46.3)
Preconception Counseling	Yes	48 (50.5)
	No	47 (49.5)
Parity	Nulliparous	50 (52.6)
	Primiparous	33 (34.7)
	Multiparous	12 (12.7)
Outcome of Previous Pregnancies *	Abortion	10 (10.5)
	Stillbirth	2 (2.1)
	Live birth	44 (46.3)
	Other	2 (2.1)
Family History of Congenital Defects	Yes	16 (16.8)
	–Congenital heart defects	8 (8.4)
	–Nervous system defects	6 (6.3)
	–Skeletal defects	2 (2.1)
	–Renal defects	1 (1.1)
	No	79 (83.2)
Preconception BMI	Normal weight (including underweight *)	57 (60.0)
	Overweight	27 (28.4)
	Obesity	11 (11.6)

Abbreviations: BMI, body mass index. * Underweight participants (***n*** = 1) were grouped with normal-weight women for analytical purposes due to the very small sample size.

**Table 2:** Bivariate logistic regression analysis of predictors of preconception folic acid supplementation (*n* = 95).

Variable	OR	95% CI	*p*-Value
Age (per year)	1.09	1.00–1.19	0.046
BMI (per kg/m^2^)	1.070	0.96–1.19	0.205
Place of residence (urban vs. rural)	1.02	0.29–3.51	0.979
Educational level (higher vs. college/school)	1.94	0.65–5.84	0.238
Planned pregnancy (yes vs. no)	9.7	1.22–76.25	0.031
Preconception counseling (yes vs. no)	7.7	2.37–24.85	0.001
Multigravida (vs. primigravida)	2.44	0.92–6.42	0.072
Previous FA supplementation (yes vs. no)	3.11	1.20–8.33	0.020

Abbreviations: OR—odds ratio; CI—confidence interval; FA—folic acid. *p* < 0.05 was considered statistically significant.

**Table 3:** Bivariate logistic regression analysis of predictors of post-conception folic acid supplementation (*n* = 95).

Variable	OR	95% CI	*p*-Value
Age (per year)	1.01	0.92–1.11	0.821
BMI (per kg/m^2^)	0.97	0.68–5.36	0.222
Place of residence (urban vs. rural)	1.42	0.40–5.03	0.585
Educational level (higher vs. college/school)	0.54	0.16–1.81	0.321
Planned pregnancy (yes vs. no)	1.16	0.34–3.96	0.808
Preconception counseling (yes vs. no)	1.91	0.68–5.36	0.222
Multigravida (vs. primigravida)	0.90	0.33–2.46	0.837
Previous FA supplementation (yes vs. no)	4.1	1.10–14.29	0.036

Abbreviations: OR—odds ratio; CI—confidence interval; FA—folic acid. *p* < 0.05 was considered statistically significant.

**Table 4:** Participants’ knowledge about periconceptional FA supplementation.

Knowledge Item	Total (*n* = 95) *n* (%)	Higher Education (*n* = 66) *n* (%)	College/School (*n* = 29) *n* (%)	*p*-Value *
Is it necessary to take FA before pregnancy?				0.03
Yes	34 (35.8)	27 (40.9)	7 (24.1)	
No	9 (9.5)	3 (4.6)	6 (20.7)	
Do not know	52 (54.7)	36 (54.6)	16 (55.2)	
Recommended duration of FA intake before pregnancy				0.03
≤1 month	5 (5.3)	5 (7.6)	0 (0.0)	
1–2 months	10 (10.5)	5 (7.6)	5 (17.2)	
≥2 months	6 (6.3)	5 (7.6)	1 (3.5)	
≥2 months and throughout attempts to conceive	13 (13.7)	12 (18.2)	1 (3.5)	
Do not know	52 (54.7)	36 (54.6)	16 (55.2)	
Is it necessary to take FA during pregnancy?				0.31
Yes	46 (48.4)	34 (51.5)	12 (41.4)	
No	3 (3.2)	1 (1.5)	2 (6.9)	
Do not know	46 (48.4)	31 (47.0)	15 (51.7)	
Recommended duration of FA intake during pregnancy				0.63
≤1 month	3 (3.2)	2 (3.0)	1 (3.5)	
1–2 months	15 (15.8)	11 (16.7)	4 (13.8)	
≥2 months	25 (26.3)	19 (28.8)	6 (20.7)	
Do not know	49 (51.6)	33 (50.0)	16 (55.2)	

Abbreviations: FA, folic acid. * *p*-values were calculated using the chi-square test comparing women with higher education versus college/school education. A *p*-value < 0.05 was considered statistically significant.

## Data Availability

The original contributions presented in this study are included in the article. Additional data are not publicly available due to privacy and ethical restrictions. Further inquiries can be directed to the corresponding author.

## References

[1] Karsonovich T., Munakomi S. (2025). Spina Bifida. StatPearls [Internet].

[2] Bazylevych A., Tychkivska O., Yevtushok L., Wertelecki W. (2016). Epidemic of neural tube defects in Ukraine, or why children’s death and disabilities are not prevented. Proc. Shevchenko Sci. Soc. Med. Sci..

[3] Salih M.A., Murshid W.R., Seidahmed M.Z. (2014). Classification, clinical features, and genetics of neural tube defects. Saudi Med. J..

[4] Pektaş A., Boyacı M.G., Koyuncu H., Pektaş M.K., Kundak A.A. (2021). Timeliness of postnatal surgery in newborns with open neural tube defects: A single center experience. Turk. J. Pediatr..

[5] Boyarchuk O.R., Koshmaniuk M.V., Hlushko K.T., Lovga M.I., Savkiv D.V. (2023). Spina bifida health issues of children in Ukraine. Modern Pediatr. Ukraine.

[6] Boyarchuk O.R., Koshmaniuk M.V. (2023). The program of multidisciplinary online support of children with spina bifida in Ukraine during the war. Child’s Health.

[7] Morris J.K., Addor M.C., Ballardini E., Barisic I., Barrachina-Bonet L., Braz P., Cavero-Carbonell C., Den Hond E., Garne E., Gatt M. (2021). Prevention of Neural Tube Defects in Europe: A Public Health Failure. Front. Pediatr..

[8] Çaylan N., Yalçin S.S., Tezel B., Aydin Ş., Üner O.K.F. (2022). Evaluation of neural tube defects from 2014 to 2019 in Turkey. BMC Pregnancy Childbirth.

[9] Blencowe H., Kancherla V., Moorthie S., Darlison M.W., Modell B. (2018). Estimates of global and regional prevalence of neural tube defects for 2015: A systematic analysis. Ann. N. Y. Acad. Sci..

[10] Dobrovolska L.I., Boyarchuk O.R., Kinash M.I. (2022). Dietary intake of folate and the frequency of its deficiency in children with type 1 diabetes mellitus and healthy children. Ukr. Biochem. J..

[11] Abate B.B., Kumsa H., Kibret G.A., Wodaynew T., Habtie T.E., Kassa M., Munie M.A., Temesgen D., Tilahun B.D., Merchaw A. (2025). Preconception Folic Acid and Multivitamin Supplementation for the Prevention of Neural Tube Defect: An Umbrella Review of Systematic Review and Meta-analysis. Neuroepidemiology.

[12] Wertelecki W., Ievtushok B., Zymak-Zakutnia N., Kalynka S., Korzhynskyy Y., Lapchenko S., Sosyniuk Z. (2016). Birth defects, Polissia, Chornobyl. Neonatol. Surg. Perinat. Med..

[13] Wertelecki W., Yevtushok L., Kuznietsov I., Komov O., Lapchenko S., Akhmedzanova D., Ostapchuk L. (2018). Chornobyl, radiation, neural tube defects, and microcephaly. Eur. J. Med. Genet..

[14] van Rooij D., Mou Y., White T., Voortman T., Jansen P.W., Buitelaar J.K. (2025). Prenatal Vitamin D, Multivitamin, and Folic Acid Supplementation and Brain Structure in Children with ADHD and ASD Traits: The Generation R Study. Nutrients.

[15] Peng L., Gao Y., Yuan C., Kuang H. (2025). Maternal red blood cell folate and vitamin B metabolism with pregnancy outcomes: A retrospective study. BMC Pregnancy Childbirth.

[16] World Health Organization (2015). Guideline: Optimal Serum and Red Blood Cell Folate Concentrations in Women of Reproductive Age for Prevention of Neural Tube Defects.

[17] CDC (1992). Recommendations for the use of folic acid to reduce the number of cases of spina bifida and other neural tube defects. MMWR Recomm Rep..

[18] National Institute for Health and Care Excellence (2025). Maternal and Child Nutrition: Nutrition and Weight Management in Pregnancy, and Nutrition in Children up to 5 Years. https://www.nice.org.uk/guidance/ng247/resources/maternal-and-child-nutrition-nutrition-and-weight-management-in-pregnancy-and-nutrition-in-children-up-to-5-years-pdf-66143961638341.

[19] Linnell A., Murphy N., Godwin J., Cremona A. (2022). An evaluation of adherence to folic acid supplementation in pregnant women during early gestation for the prevention of neural tube defects. Public Health Nutr..

[20] Medawar G., Wehbe T., Jaoude E.A. (2019). Awareness and Use of Folic Acid among Women of Childbearing Age. Ann. Glob. Health.

[21] Köken G.N., Derbent A.U., Erol O., Saygın N., Ayık H., Karaca M. (2013). Awareness and use of folic acid among reproductive age and pregnant women. J. Turk. Ger. Gynecol. Assoc..

[22] Nilsen R.M., Leoncini E., Gastaldi P., Allegri V., Agostino R., Faravelli F., Ferrazzoli F., Finale E., Ghirri P., Scarano G. (2016). Prevalence and Determinants of Preconception Folic Acid Use: An Italian Multicenter Survey. Ital. J. Pediatr..

[23] Yamamoto S., Wada Y. (2018). Awareness, use and information sources of folic acid supplementation to prevent neural tube defects in pregnant Japanese women. Public Health Nutr..

[24] Jin Y.J., Kim H.W. (2023). Influence of folic acid knowledge on effective folic acid intake in Chinese pregnant women: A cross-sectional study. Korean J. Women Health Nurs..

[25] Teshome F., Kebede Y., Girma K., Birhanu Z. (2022). A survey on women’s awareness of iron and folic acid intake during preconception period and its associated factors in Manna District, Oromia region, Southwest Ethiopia. Nurs. Open.

[26] Zadarko-Domaradzka M., Kruszyńska E., Zadarko E. (2021). Effectiveness of Folic Acid Supplementation Recommendations among Polish Female Students from the Podkarpackie Region. Nutrients.

[27] Hlushko K., Boyarchuk O., Kinash M., Burbela E., Rohalska Y., Dobrovolska L. (2021). Awareness of folic acid use and its effects among medical students in Ukraine. Wiad. Lek..

[28] Panasiuk A., Hozyasz K.K. (2023). Knowledge and supplementation of folic acid among female university students in southeastern Poland. Actual Gyn..

[29] Bitzer J., von Stenglin A., Bannemerschult R. (2013). Women’s awareness and periconceptional use of folic acid: Data from a large European survey. Int. J. Womens Health.

